# Upregulation of the Oct3/4 Network in Basal Breast Cancer Is Associated with Its Metastatic Potential and Shows Tissue Dependent Variability

**DOI:** 10.3390/ijms24119142

**Published:** 2023-05-23

**Authors:** Robin G. Rajan, Raisa I. Krutilina, Tatyana N. Ignatova, Zoran S. Pavicevich, Galina M. Dulatova, Maria A. Lane, Arindam R. Chatterjee, Robert J. Rooney, Mymoon Antony, Vivian R. Hagerty, Nickolay V. Kukekov, Khalid A. Hanafy, Frank D. Vrionis

**Affiliations:** 1 Helene and Stephen Weicholz Cell Therapy Laboratory, Marcus Neuroscience Institute, 800 Meadows Road, Boca Raton, FL 33486, USA; 2 Department of Pathology and Laboratory Medicine, Center for Cancer Research, University of Tennessee Health Sciences Center, 920 Madison Ave., Memphis, TN 38163, USA; 3 Mallinckrodt Institute of Radiology, Departments of Neurosurgery and Neurology, Washington University School of Medicine, 660 S. Euclid Ave., St. Louis, MO 63110, USA; 4 Le-Bonheur Children’s Outpatient Hospital, 51 N Dunlap St., Memphis, TN 38105, USA; 5 Wellington Regional Medical Center, 10101 Forest Hill Blvd, Wellington, FL 33414, USA; 6 Department of Biomedical Science, Schmidt College of Medicine, Florida Atlantic University, 777 Glades Road, Boca Raton, FL 33431, USA

**Keywords:** breast cancer, metastasis, oct3/4 transcriptome, cancer stem cells, MDA-MB-231

## Abstract

Adaptive plasticity of Breast Cancer stem cells (BCSCs) is strongly correlated with cancer progression and resistance, leading to a poor prognosis. In this study, we report the expression profile of several pioneer transcription factors of the *Oct3/4* network associated with tumor initiation and metastasis. In the triple negative breast cancer cell line (MDA-MB-231) stably transfected with human Oct3/4-GFP, differentially expressed genes (DEGs) were identified using qPCR and microarray, and the resistance to paclitaxel was assessed using an MTS assay. The tumor-seeding potential in immunocompromised (NOD-SCID) mice and DEGs in the tumors were also assessed along with the intra-tumor (CD44+/CD24-) expression using flow cytometry. Unlike 2-D cultures, the Oct3/4-GFP expression was homogenous and stable in 3-D mammospheres developed from BCSCs. A total of 25 DEGs including *Gata6, FoxA2, Sall4, Zic2, H2afJ, Stc1* and *Bmi1* were identified in Oct3/4 activated cells coupled with a significantly increased resistance to paclitaxel. In mice, the higher Oct3/4 expression in tumors correlated with enhanced tumorigenic potential and aggressive growth, with metastatic lesions showing a >5-fold upregulation of DEGs compared to orthotopic tumors and variability in different tissues with the highest modulation in the brain. Serially re-implanting tumors in mice as a model of recurrence and metastasis highlighted the sustained upregulation of *Sall4, c-Myc, Mmp1, Mmp9* and *Dkk1* genes in metastatic lesions with a 2-fold higher expression of stem cell markers (CD44+/CD24-). Thus, *Oct3/4* transcriptome may drive the differentiation and maintenance of BCSCs, promoting their tumorigenic potential, metastasis and resistance to drugs such as paclitaxel with tissue-specific heterogeneity.

## 1. Introduction

Metastasis in patients with breast cancer (BC) significantly increases morbidity and mortality [1,2]. However, the underlying mechanisms driving metastasis are not fully understood. A prominent obstacle to effective treatment is the intra-tumor cellular and molecular heterogeneity promoted by genetic, epigenetic and micro environmental factors [3].

Minor pools of cancer stem cells (CSC) and the epithelial–mesenchymal transition (EMT) determine tumor recurrence, growth, metastasis and resistance to chemotherapy [4]. Several similarities exist in the molecular signature between cultured human embryonic stem cells (hES) and CSCs, including the developmental regulatory network of the pioneer transcriptional factors (PTFs) *Oct3/4, Nanog, Sox2* and *Sall4* (ONSS network). Oct3/4 inhibits differentiation and is primarily involved in the maintenance of the pluripotency of stem cells. During embryogenesis, cellular transcriptional profiles of ES cells in the blastula inner cell mass are constantly re-assorted by PTFs, including the ONSS network along with *FoxA2*, and *Gata6,* which are crucial for the activation of genes in compact chromatin [5,6]. Such transcriptional complexes form gene regulatory networks that consistently maintain transcriptional control during embryogenesis and underlie the mechanism of epigenetic memory required for the optimal expression of these genes under normal growth conditions [7]. Most fatal malignancies demonstrate aberrantly activated PTFs, such as *Sox2, Nanog* and *Sall4;* however, the complex regulatory mechanism responsible for selective organ specific metastasis for most types of tumors remains poorly understood. Although several pathways contributing to metastasis have been identified [8,9,10], the causal role of the transcriptional plasticity of BCSCs and their specificity for different organs in metastasis needs elucidation.

The current concept of oncogenic reprogramming explains oncogenesis as an interplay between PTFs and chromatin regulators, which is essential for the maintenance of CSCs’ dynamic plasticity. Growing evidence demonstrates the role of growth conditions in determining the functional cellular plasticity in BC cells [11]. It remains unclear whether growth constraints or stress induce epigenetic changes as an adaptive response, or whether they inadvertently select the most resistant clone and thus promote growth and metastasis. It has been recently demonstrated that only a small subset of pre-existing competent cells can adapt to unfavorable growth conditions by transcriptional modulation in an epigenetic manner [12]. Currently, the link between a regulatory mechanism of CSC’s stemness and oncogenic developmental is not yet clear [13,14].

CSCs in primary tumor biopsies are rare and spontaneous differentiation makes it difficult for the conventional 2D cell culture to enrich and maintain these CSC. There is a need for a model culture system to stably propagate CSCs with sustained stem cell characteristics. We have previously utilized a plasmid with the promoter for the human Oct3/4 gene tagged with a GFP reporter (phOCT4p-eGFP) and demonstrated its use to identify undifferentiated self-renewing hES [15], isolate tumorigenic cells from osteosarcoma cell populations [16], and highlight the role of Oct3/4 in regulating stemness in CSCs and tumor initiation in Glioblastoma (GBM) [17]. The modulation of several genes in the Oct3/4 pathway was associated with epigenetically directing oncogenic reprogramming, cellular physiology, and metabolism during tumor development in cervical and gastric cancers [18,19]. Our current study aimed to assess the role of transcription factors including ONSS and other related genes in the Oct3/4 network to regulate the tumorigenicity of breast cancer stem cells and drive their organ-specific metastatic potential using the MDA-MB-231 breast cancer cell line. We demonstrated the ability of BCSCs to selectively activate key pioneer transcription factors and other genes of the Oct3/4 pathway in different metastatic tissue niches, inducing tumor growth and treatment resistance.

## 2. Results

### 2.1. Oct3/4-Transfected MDA-MB-231 Cells Demonstrate Positive Correlation with Oct3/4 Expression and Cancer Stem Cell Phenotype with an Upregulated Transcriptome of Pioneer Factors

**Figure 1:**
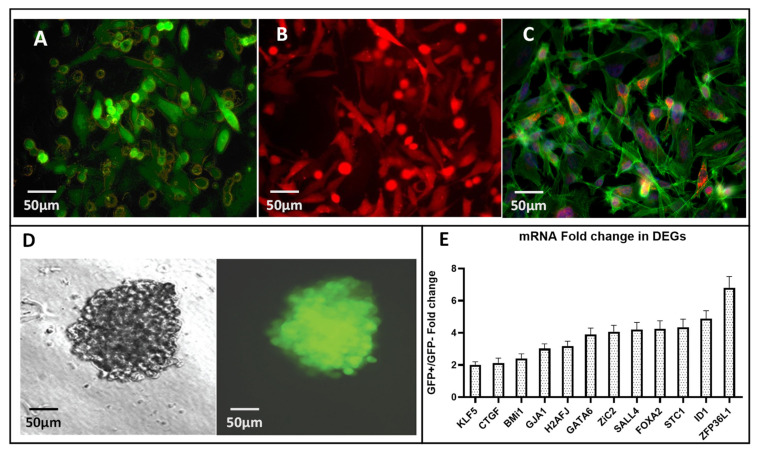
In vitro identification and selection of Oct3/4-transfected MDA-MB-231 cells with differentially expressed genes. (**A**–**C**) MDA-MB-231 cells co-transfected with Oct3/4-GFP and CMV-mRFP were identified using green and red fluorescence, respectively. (**A**) GFP-only positive and negative cells. (**B**) RFP-only positive and negative cells. (**C**) GFP-RFP double positive and negative cells were identified as distinct populations. (**D**) Mammary epithelial stem cell aggregates (mammospheres) generated from monoclonal GFP+ cells demonstrate stable and homogenous Oct3/4-GFP expression when assessed by fluorescence microscopy. (**E**) Using qPCR and microarray analysis of the transfected in vitro cultures, mRNA level of several differentially expressed genes (DEG) was assessed. Gene expression in (RFP+GFP+) cells when compared to (RFP+ GFP-) cells is presented as fold change. Data presented as mean ± SD is from at least 3 different cultures tested in triplicate, and DEG significance is per Student’s *t*-test with *p* < 0.001–0.01.

The triple negative BC cell line MDA-MB-231 was co-transfected with an exogenous GFP-tagged OCT3/4 promoter (phOCT4p-eGFP) and RFP-tagged CMV promoter (phCMV-mRFP). These cells were cultured in optimal growth conditions for the stable expression of *Oct3/4,* as described in the methods. The CMV-mRFP plasmid was utilized to determine the transfection efficiency, and we isolated stably transfected cells using GFP and RFP fluorescence as well as G418 antibiotic resistance. Three distinct populations, namely (GFP+RFP+), (GFP+RFP-) and (GFP-RFP+), could be visualized (Figure 1A–C) and were sorted using FACS. Using growth conditions specific for human embryonic stem cells (ESCs), as previously described [17,20], GFP+ and GFP− clones were cultured separately in a stem cell culture (SCC) medium. Single cells were assessed for their ability to maintain a stable expression of the *Oct3/4-*GFP reporter after replication in a 2D culture in normal growth media and in 3D mammospheres (mammary epithelial stem cell aggregates) cultured in SCC media. Two-dimensional cultures demonstrated a polyclonal phenotype with respect to the *Oct3/4*-GFP expression (Figure 1A–C, Supplementary Figure S1), while mammospheres cultured in the SCC medium demonstrated a stable expression of transfected Oct3/4 assessed by GFP fluorescence (Figure 1D), indicating a direct correlation with the Oct3/4 expression and stem cell phenotype of the transfected MDA-MB-231 cells. Several such clones assessed by flow cytometry revealed intra-clonal heterogeneity in the GFP expression (Supplementary Figure S1). A randomly selected clone (Clone#9, Supplementary Figure S1) with its (GFP+) and (GFP-) counterparts were selected using FACS for further in vitro genetic analysis and in vivo tumor growth studies. Using a qPCR and microarray analysis of the transfected MDA-MB-231 cells, FACS-purified (GFP+) cells were assessed for differentially expressed genes (expression change >2 log-fold, *t*-test *p* value < 0.01) when compared to (GFP- cells). We focused on several pioneer transcription factors and growth and developmental regulators of the Oct3/4 pathway that are involved in diverse cellular functions [21]. Developmental regulators, strongly associated with the early endo-mesodermal and neural cell-fate determination at gastrulation, such as *Gata6*, *Sall4, Zic2, Bmi1, Klf5* and *FoxA2*, demonstrated a 2–4-fold upregulation. *H2AfJ*,a *J*-variant of the core H2A histone family and a group of genes involved in maintaining the cellular architecture, such as the actin cytoskeleton rearrangement required for EMT and MET transition (*connexin-43/Gja1*), as well as *Id1,* also demonstrated a significant modulation in expression. The early growth response regulator *ZFP36L1* demonstrated the highest (8-fold) increase in mRNA expression (Figure 1E).

The significant differential gene expression was determined by a Welch‘s *t*-test, using a *p*-value of <0.01 and a >2-fold expression change. Based on these criteria, 17 genes exhibited a significant differential expression (Differentially Expressed Genes or DEGs, *p ≤* 0.0001–0.01) in the analyzed samples. The results were confirmed by real-time reverse transcription PCR.

### 2.2. Oct3/4 Expression Is Associated with High Tumorigenic Potential of MDA-MB-231 Cells 

**Figure 2:**
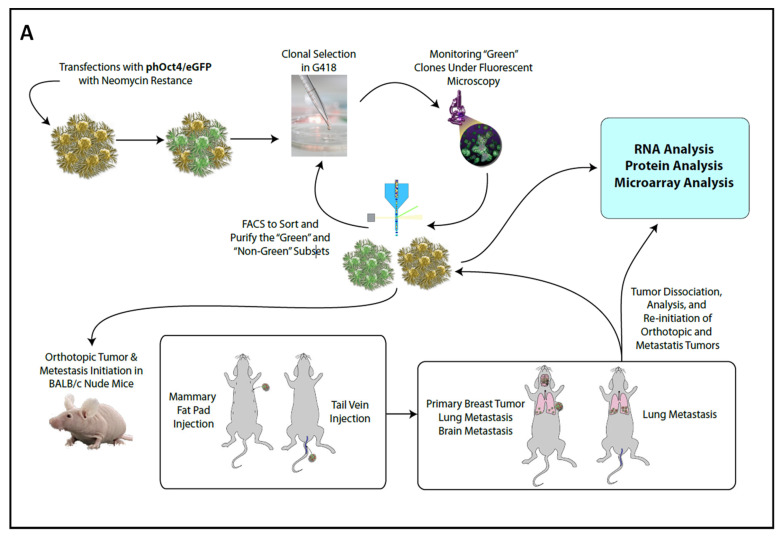

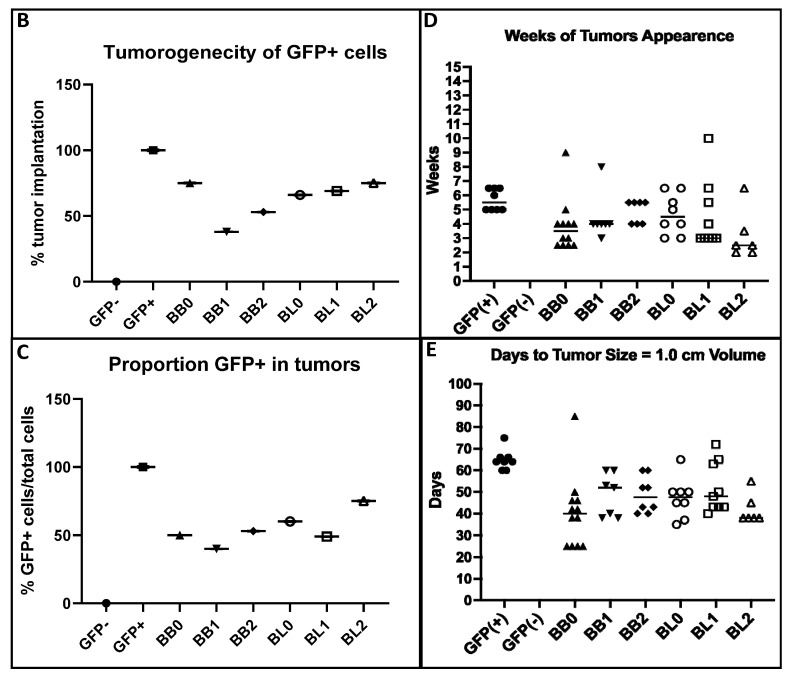
Mouse tumor-seeding potential of Oct3/4-transfected MDA-MB-231 cells. (**A**) Experimental paradigm (orthotopic and metastatic model) used in this study. (**B**–**E**) Oct3/4-GFP transfected MDA-MB-231 cells were implanted SQ in mammary fat pad of nude mice and assessed for tumor-forming potential. (**B**) Comparison of successful tumor implantation by transfected GFP+ and GFP- cells in breast (multiple rounds of re-implantation indicated by BB0-BB2) and corresponding lung metastasis (BL0-BL2). (**C**) Proportion of GFP+ cells in the tumors during each round of implantation measured by flow cytometry and comparison of tumor aggressiveness indicated by (**D**) time taken to establish tumors and (**E**) time for tumor to reach 1 cm. GFP+ and GFP−: Primary orthotopic breast tumor in the mammary fat pad by transfected MDA-MB-231 cells. BB0, BB1 and BB2: Breast tumors from the serial re-implant of primary tumors by GFP+ cells. BL0, BL1 and BL2: Lung metastasis from BB0, BB1 and BB2, respectively. Data presented as mean ± S.D for BB0–BB2, BL0–BL2, *n* = 9 for GFP− and *n* = 8 GFP+.

We assessed the tumor-seeding capacity of MDA-MB-231(Oct3/4-GFP)+ cells in an orthotopic BALB/c nude (NOG) mouse model, as previously described [22] (Figure 2A). We orthotopically implanted FACS-purified MDA-MB-231 GFP(+) and GFP(-) cells in mice and assessed their potential to establish tumor and lung metastasis. Cells with an activated Oct3/4 reporter (GFP+) were able to generate a subcutaneous tumor with 100% efficiency, while GFP− cells had no tumor-seeding potential (Figure 2B: *n* = 9 GFP(−) group; *n* = 8 GFP+ group). The experiment was repeated three times with separate clones and demonstrated similar results.

As a model of recurrence and metastasis, tumors from the primary breast tumor and lung metastasis were dissociated and re-implanted in the mammary fat pad. Breast tumors generated from serial rounds of the re-implantation of primary breast tumor cells and corresponding lung metastatic cells into the mammary fat pad of NOG mice were termed as BB0–BB2 and BL0–BL2, respectively. We observed a significantly diminished tumor-seeding potential by primary breast tumor cells by 25% in the first round and 61% in the second round and comparatively improved the efficiency in the third round (reduced by 47%) (Figure 2B: BB0, *n* = 16; BB1, *n* = 18; BB2, *n* = 15). In the case of re-implanted lung metastatic cells in the mammary fat pad, we observed a 33% reduction in the tumor-seeding efficiency in the first round when compared to the initial SQ implant (GFP+); however, there was no more reduction in seeding potential in the subsequent rounds (Figure 2B: round1 = BL0 (*n* = 12), round2 = BL1 (*n* = 13) and round 3 = BL2 (*n* = 8).

Using flow cytometry, we also measured the proportion of GFP+ cells in each round of re-implantation and observed a positive correlation between the total percentage of Oct3/4-activated tumor cells and efficiency of tumor implantation. We observed that the reduced tumor implantation efficiency during serial rounds of re-implantation in the breast (BB0 and BB1) correlated with less GFP+ cells in the tumor, and the increase in GFP+ cells in BB2, BL0 and BL2 corresponds with a higher tumor efficiency (Figure 2B,C). Although the aggressiveness in terms of tumor growth was not significantly different, a notable difference was observed in growth kinetics of BL2 and the corresponding percentage of GFP+ cells in the tumor (Figure 2D–E). 

Although they are less efficient in establishing tumors, serially re-implanted tumors (BB0-BB1) demonstrated faster growth kinetics when compared to the very first round (GFP+) by 7–17 days, but no significant difference was observed between growth kinetics of primary breast tumor cells or metastatic lung tumor cells (Figure 2D,E). Overall, activated *Oct3/4* in MDA-MB-231 cells show a strong association with tumor initiation and lung metastasis.

### 2.3. Metastatic Potential of MDA-MB-231 Tumor Cells Is Associated with an Upregulated Oct3/4 Network Specific for Cancer Stem Cell Phenotype and Tumor Growth

**Figure 3:**
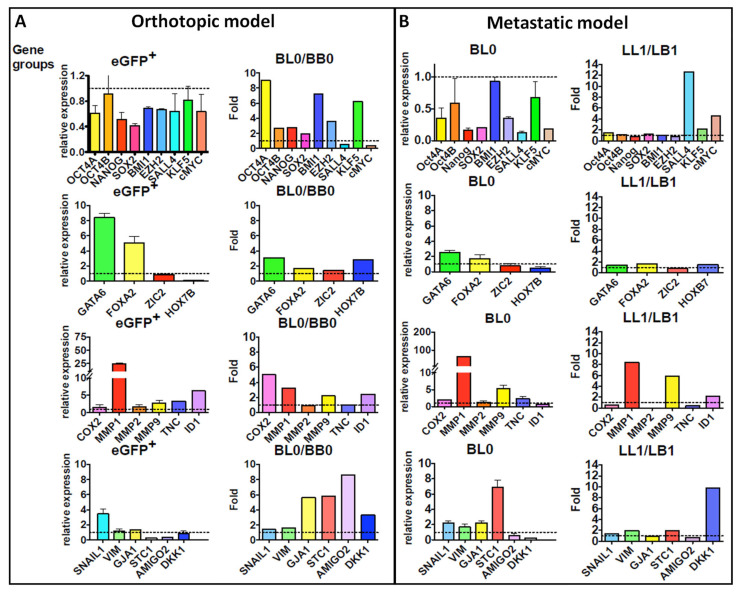
Assessing modulation of *Oct3/4* network PFs and other TFs in our orthotopic and metastatic mouse model using Oct3/4-transfected MDA-MB-231 cells. MDA-MB-231 cells transfected with Oct3/4-GFP were used to generate tumors in nude mice in breast and lungs. Comparative qRT-PCR analyses of 25 differentially expressed genes of the Oct3/4 network is presented as relative expression to housekeeping gene (GAPDH) or fold change of GFP+ cells with respect to GFP- cells in (**A**) orthotopic model and (**B**) metastatic model. The dash line indicates a relative expression of 1 as a baseline for comparison in all the graphs. eGFP+: MDA-MB-231 GFP+ cells (baseline). BB0: Cells’ primary orthotopic breast tumor from the mammary fat pad. BL0: Lung mets from the primary breast tumor, BB0. LB1: Breast tumor generated from the serial implant (round 2) of the lung mets of BL0. LL1: Lung metastasis from LB1.

In the orthotopic mouse model, we assessed the differentially expressed genes of the Oct3/4 network in spontaneous lung metastasis compared to the SQ mammary fat pad tumors using MDA-MB-231 cells transfected with the Oct3/4-GFP promoter. In the SQ breast tumors, we observed significantly upregulated mRNA levels of the Oct3/4 regulatory network, including *ONSS*, and several other PTFs, including *Nanog, Sall4, Sox2, Sox4, FoxA2, Gata6, Zic2* and *HoxB7,* as well as *Oct3/4 isoform B,* polycomb suppressors *Bmi1, EzH2, Amigo* and *Dkk1* (Figure 3A, left panel). Furthermore, the metastatic potential (lung mets-BL0) of these tumors was associated with a 2–10-fold up-regulation of these genes compared to the mRNA levels in SQ orthotopic tumors (BB0). In contrast, *Sall4, cMYC, Gata6, FoxA2, Zic2, Mmp1, Snail1* and *Vim* were downregulated in metastatic lung lesions (Figure 3A, right panel).

To further explore the modulation of this transcriptome’s association with the metastatic potential and recurrence in vivo, we sequentially re-implanted initial metastatic lung lesions back into the mammary fat pad and isolated the resultant xenograft SQ tumors (LB1–LB3) and corresponding new metastatic lung lesions (LL1–LL3) (Figure 3B). These LB1–LB3 and LL1–LL3 xenografts consistently maintained the transfected Oct3/4-GFP expression. In contrast to the orthotopic model, the second round of metastatic lung lesions (LL1) demonstrated the selective upregulation of *Sall4, Klf5, cMYC, Mmp1, Mmp9, Id1, Vim, Stc1* and *Dkk1* compared to its SQ mammary fat counterpart (LB1) (Figure 3B). Thus, metastasis demonstrates the adaptive reshuffling of *Oct3/4* and its associated transcriptome.

### 2.4. Oct3/4-Transfected MDA-MB-231 Tumors in Mice Demonstrate Organ-Specific Heterogeneity in Upregulation of Oct3/4 Transcriptome

To assess the role of tissue niches in the tumor initiation and metastatic potential of BCSCs, we evaluated the Oct3/4-related transcriptome in tumors from the mammary fat pad, lungs and brain. 

**Figure 4:**
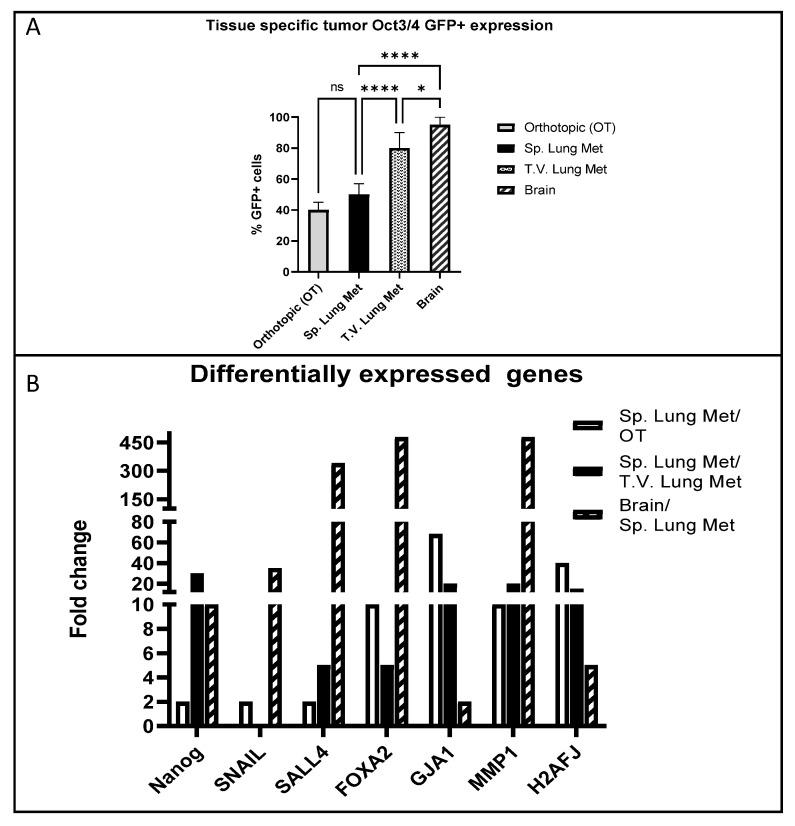
Heterogeneity of Oct 3/4 GFP expression and related transcriptome within primary tumors and metastatic lesions in different tissues. (**A**) Within tumors generated from Oct3/4-GFP-transfected MDA-MB-231 cells, proportion of Oct3/4 GFP+ cells is compared between primary tumor and metastatic lesions in different tissues. Subcutaneously implanted mammary fat pad tumor (orthotopic or OT) or lung metastasis from OT breast (sp. lung met) or lung metastasis from direct tail vain injection of cells (T.V. lung mets) or tumors formed by direct implantation in the brain (brain) were assessed for GFP+ cells using flow cytometry. Differences between the groups were analyzed using one-way ANOVA and post-Tukey multiple comparison test (data presented as mean ± S.D, *n* = 5, * *p* < 0.05, **** *p* < 0.0001, ns = not significant). (**B**) qRT-PCR analysis of differentially expressed genes of the OCT3/4 network in the GFP+ cells compared to GFP- cells within primary tumors and metastasis in different tissues. Fold changes with respect to the housekeeping genes presented as a ratio of spontaneous lung metastasis (sp. lung met), orthotopic (OT) and brain. Graph represents significantly (*p* < 0.001) upregulated genes from 3 independent cultures conducted in triplicate. (All other 36 genes assessed are included in Supplementary Figure S2).

We implanted Oct3/4-transfected BCSCs by various modes in mice, namely (1) SQ in mammary fat pad, (2) IV via tail vein and (3) orthotopically in the brain and assessed the expression of Oct3/4 in the resultant tumors. We found that tumors from the mammary fat pad and the corresponding lung metastatic lesions were comprised of 40–50% Oct3/4-GFP+ cells. The overall Oct3/4 expression was maintained significantly higher in brain tumors from direct implantation (90%) and lung tumors from IV implantation (80%) (Figure 4A).

Next, we evaluated the differentially expressed Oct3/4-related transcriptome in these tumors. We found the tumors in the brain significantly favored the upregulation of the following genes compared to lung lesions and SQ mammary fat pad tumors: *Snail, Sall4, FoxA2* and *Mmp1.* The set of genes favoring lung lesions to the brain were: *Nanog, Gja1 and H2afj* (Figure 4B). These results indicate a heterogeneous and organ-specific preferential upregulation of specific genes in the Oct3/4 network.

### 2.5. MDA-MB-231 with Activated Oct3/4 Cells Evade Paclitaxel-Induced Cell Death and Generate Tumors with Significantly Upregulated Stemness Genotype (CD44+/CD24-)

**Figure 5:**
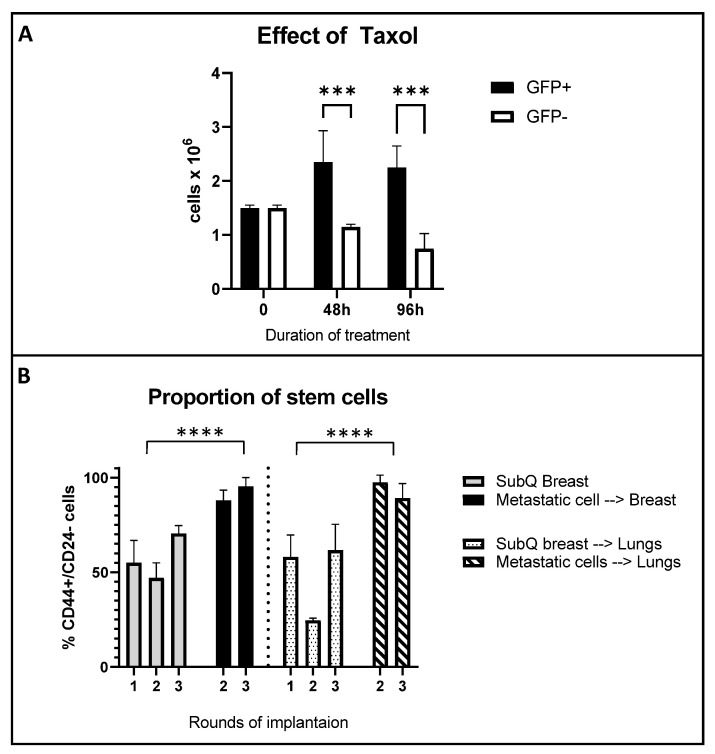
Effect of taxol on proliferation of MDA-MB-231 cells with activated *Oct3/4* and assessment of tumor stemness in metastatic cells. (**A**) Oct3/4-GFP-transfected MDA-MB-231 cells cultured in standard growth media were treated with paclitaxel for 48 h or 96 h and assessed for proliferation. Differences in proliferation between GFP+ and GFP- cells is presented as total cells remaining after the treatment period. Results from three independent cultures performed in triplicate. (**B**) Flow cytometry assessment of stemness by quantifying CD44+/CD24- expression in tumors from mammary fat pad and lung metastases. Multiple rounds of tumor implantation follow our orthotopic and metastasis model, as explained in the methods. Differences between the groups were analyzed using one-way ANOVA and post-Dunnett’s multiple comparison test (data presented as mean ± S.D, *n* = 5 for tumor studies, *** *p* < 0.0002 and **** *p* < 0.0001). Metastatic cell→ Breast: indicate SQ tumors in mammary fat pad (round 2) by re-implanting lung mets from round 1. SQ breast→ Lungs: Indicate spontaneous lung mets from SQ breast tumor in round 1. Metastatic cell→ Lungs: Indicate spontaneous lung mets from “Metastatic cell→ Breast“ tumors (round 2).

To determine the resistance of BCSC to standard chemotherapy, we evaluated the effect of paclitaxel on the proliferation of MDA-MB-231 cells with an activated Oct3/4-GFP promoter. Using a proliferation assay, we measured the survival and growth of MDA-MB-231 GFP+ and GFP− cells treated with 10 nMPaclitaxel for up to 96 h. The concentration of paclitaxel (1C50 of 10 nM) and time for the study was based on previously established studies of effects of paclitaxel on the basal MDA-MB-231 cell line [23]. We found that cells with activated *Oct3/4* demonstrated significantly better survival and proliferation compared to GFP- cells (1.2–1.9 fold, *p* < 0.01, *n* = 5) after 48 h and 96 h of treatment (Figure 5A). The comparative difference in the proliferation of GFP+ cells indicate a strong association of the activation of the *Oct3/4* network and treatment resistance.

The CD44+ genotype in primary breast tumors and metastasis has been positively associated with tumor initiation and progression, metastasis and increased stemness [24], whereas CD44+/CD24- is considered a hallmark of BC stem cells. To assess the CSC property within the tumor cells, we used flow cytometry to determine the CD44 and CD24 distribution in the primary orthotopic tumor (SQ mammary fat) and metastatic lung lesions. Overall, the proportion of (CD44+/CD24-) stem cells in the SQ mammary fat pad tumors was only slightly enriched after the third round of serial re-implantation (50% to 70%), with no change in the corresponding lung metastatic lesions in each round (Figure 5B, grey bars). However, metastatic lung lesions, when re-implanted orthotopically in the mammary fat pad, or if progressed to the lung as metastatic lesions, demonstrated a significantly higher proportion of stem cells (90–100%) and even maintained their complete stemness phenotype after rounds of sequential re-implantation (Figure 5B, dark and checkered bars).

## 3. Discussion

The metastatic potential of breast cancer significantly correlates with treatment failures, leading to increased morbidity and mortality, and is strongly mediated by CSCs [25]. Although causal mechanisms of PTFs such as Oct3/4 in cancer are complex and difficult to unravel, their differential expression in breast cancer compared to normal tissues is well established [26]. Several studies have revealed the role of Oct3/4-regulated PTFs in maintaining pluripotency, differentiation and EMT/MET plasticity in several types of cancers, including breast cancer [18,19]. In this study, we explored the association of a Oct3/4-related network of transcription factors, with the tumor initiation and metastatic potential of BCSCs. Additionally, we also assessed the tissue specific heterogeneity in the expression of this transcriptome, which may shed light on organ preferences for metastasis. For example: tumors in breast cancer have metastatic preference for bones, lungs, liver and brain [27]. We used the triple negative metastatic breast cancer cell line MDA-MB-231 transfected with an exogenous human Oct3/4-GFP promoter in a mouse tumor model. Two-dimensional cultures in a normal growth medium, both polyclonal and monoclonal, demonstrated a variable Oct3/4-GFP expression (Supplementary Figure S2). The heterogeneity of the cells in the culture with respect to the Oct3/4 activation could be modulated by growth conditions such as the addition of growth factors, anchorage dependence and serum withdrawal in the stem cell culture (SCC) media to induce and stably maintain the Oct3/4 expression. We have previously optimized the generation of brain tumor spheres with a sustained Oct3/4 expression in a glioma model and in osteosarcoma [17,20]. In this study, we were able to generate mammary epithelial stem cell aggregates (mammospheres) and verified the complete and stable expression of the Oct3/4 expression using GFP and flow cytometry (Figure 1A,B). 

The aberrantly activated Oct-3/4 and its increased expression strongly correlates to the malignant potential of germ cells in other cancers [28]. Using the qPCR and microarray analysis of the Oct3/4-GFP transfected cells, we identified 12 differentially expressed genes (DEG) compared to non-transfected tumor cells related to the Oct3/4 network (Figure 1C). A differentially upregulated transcriptomic landscape of the *Oct3/4*-transfected cells represents a collective portrait of the stem cell regulatory network. This set of DEG (Figure 1C) indicates multipotent stem cells, expressing endodermal, mesenchymal and neuroectodermal marker genes along with progenitors of CSC suggested by the expression of *Gata6*, *Sall4, Zic2, Bmi1, Klf5* and *FoxA2*. Transcription factors controlling EMT/MET, namely *Gja1* and *Id1,* as well as a growth regulator, such as *ZFP36L1,* were differentially modulated as well. These results strongly suggest that the well-known heterogeneity of cancer cells is strongly associated with the heterogeneity of the developmentally hierarchic progeny generated by multipotent BCSCs. Thus, the collective heterogeneous transcriptomic portraits of Oct3/4-transfected MDA-MB-231 cells are the cumulative results of the developmental and stochastic heterogeneity representing a stem cell phenotype specific to BCSCs. 

Furthermore, we evaluated the tumorigenesis potential of *Oct3/4*-transfected BC cells in vivo, using a nude BALB/c mouse model, and observed that the *Oct3/4* activation and proportion of *Oct3/4* expression is associated with a marked increase in the tumor-seeding potential (initiation), recurrence and metastasis compared to cells with inactive *Oct3/4* (Figure 2B–E). Serial reimplantation was not only a model of recurrence and metastasis, but we also wanted to observe the stability of the transfected Oct3/4 gene in these cells. Unlike some transcription factors, such as GATA1-4, BMI1and HMG1, the endogenous Oct3/4 usually does not show “mitotic bookmarking”, thus is not carried over after replication by default [29,30]. Clonal selection is a possibility and could have been aided by the overactivation of Oct3/4 gene as well as the related ONSS transcriptome that rendered the predisposition to metastasis and increased tumor initiation potential. We measured the proportion of Oct3/4-activated cells within the tumor using FACS (Figure 2C) and correlated the level of Oct3/4 expression with the tumorigenicity of the cells on re-implantation to understand the mechanism. This observation is concurrent with other studies, indicating an increased expression of Oct3/4 in tumor-initiating cells [31] and the upregulated transcriptome of Oct3/4-related genes, inducing stem cell characteristics in these tumor cells (Figure 1C). 

Differentially expressed transcriptome in the tumors generated from activated Oct3/4 in cancer cells indicate a multipotent stem cell signature associated with heterogeneous tumor sub-populations, including quiescent dormant cells, germ cell progenitors and cells with differentiated markers of endoderm, mesoderm (*Vimentin*, Snail-EMT genes) and neuroectoderm (*Amigo*). The upregulation of *Gja1/connexin43*, the marker of re-epithelization of metastatic cells, indicates that the bidirectional interconversion of EMT-MET is controlled by the Oct3/4-regulated ONSS (Figure 3A,B). Several studies have highlighted the role of *Myc* proto-oncogenes with *Gja1/connexin43* in the stress-associated rewiring of the gene regulatory network, increasing epigenetic plasticity in cells to make them more adaptable and promoting tumorigenesis in different tissues [32]. The Oct3/4-induced upregulation of the *c-Myc* genes may be promoting the opening of chromatin and thus inducing polyploidy in the CSC, indicating an adaptive response to the cellular stress and morphogenesis required during metastasis. It is also important to note that the upregulated Sox2 operates through transcriptional cascades, with Oct3/4 acting as a recursive series of interlocked feedforward modules maintaining the pluripotency of ES cells [33]. This reciprocal transcriptional structure results in a sequential redundancy demonstrated for ONSS genes and ensures the metastatic spread that cannot be abolished by removing a single factor or multiple alternate factors in the gene regulatory cascade. However, the elimination of two sequential factors such as Sox2 and Oct3/4 may inhibit the aggressive growth and metastatic potential of such CSCs. An example of such a strategy was highlighted for the Gata6 and *Muc1/β-Catenin* pathway in cholangiocarcinoma [34].

The tumor suppressive activity of *FoxA2* is associated with its induction of *Dkk1, Cu12* and *Cdc73* [35]. *Dkk1* has regulatory functions in the *wnt* signaling pathway and metalloproteinase expression (*Mmp-1, -2* and *-9)* and has been associated with a poor prognosis in several cancers [36]. Based on its known function in several other cancers, the differentially upregulated *Dkk1* along with *Mmp1 and Mmp9* in the MDA-MB-231 cells with an activated Oct3/4, as observed in our study, may be contributing to tumor progression by promoting migration, invasion, proliferation and enhancing cancer stem cell-like properties. Interestingly, we observe a more significant and conserved upregulation of these two factors in the metastatic lesions (lungs) when compared to the primary tumor (SQ breast) (Figure 3A,B). Oct4 is a corepressor for *FoxA2* and *Gata* homologs that specify ectodermal (*FoxA*), endodermal and mesodermal (*Gata*) differentiation [37]. Together, they direct the embryonic lineage-specific transcriptional regulation to maintain appropriate developmental timing and offer protection against aberrant tissue differentiation and thus tumor growth [38,39]. 

*Sall4* governs stemness and cell-fate through the transcriptional regulation of *Oct3/4, Nanog, Gata4, Gata6* and the *Sox* family members by forming a crucial interconnected auto-regulatory network with self-repressing feedback loops [40,41]. Sall4 is associated with recurrence and metastasis in several type of cancers, including breast cancer [42], and its suppressive action on target genes prevents cell differentiation and maintains pluripotency by engaging an epigenetic repressor, such as Mi-2/NuRD, with histone deacetylase activity (HDAC) [43], DNA methyltransferases [44], as well as *c-MYC* and *CCND1* down-stream targets of the *Wnt/beta*-catenin signaling pathway [45]. This interconnected and complex relationship between the *Oct3/4*-directed *Sall4* is influenced by the external microenvironment in different tissue niches [46], as also observed in Figure 4B with a 300-fold difference in its *Oct3/4*-directed expression in brain tumors compared to lungs and breast. Additionally, metastatic lesions in lungs maintained a *Sall4* expression at a much higher level (by 12-fold) in our metastatic model when compared to tumors in the breast; Figure 3B. Our findings, with respect to the tissue-specific modulation of *ONSS,* promoting metastasis, are supported by several previous studies [5,6] indicating the cell-type or tissue-specific factors influencing the function of the *Sall4* complexed with the Polycomb-repressive complex in the chromatin reorganization and binding bivalent domain. We observed that there was a significant heterogeneity in the expression of Oct3/4 in the tumors propagated in the brain vs. lungs or mammary fat pad. Tumors resulting from a direct implantation in the brain and lungs (via IV) demonstrated a 100% overall Oct3/4-GFP expression, while SQ mammary fat tumors and metastatic lesions had only 50% of GFP+ cells (Figure 4A). Additionally, this observation was also correlated with highest upregulation of ONSS PTFs and other related genes in the brain when compared to lung metastatic lesions and orthotopic mammary fat pad tumors, except for *Gja1* and *H2faJ* (Figure 4B). This indicates the presence of tissue-specific factors affecting the regulation of the Oct3/4-related transcriptome.

Next, we utilized flow cytometry to assess the overall stemness of the tumors generated from Oct3/4-transfected BC cells. Although CD44+/CD24- is considered a marker of BC stem cells, CD24 has been historically identified as a marker of cell signaling in tumors and recently has been reported as an anti-phagocytic surface protein on breast cancer cells. This suggests an immunosuppressive phenotype for CD24+ cells and a potential immunotherapeutic target [47]. Metastatic lung lesions had a significantly higher proportion (up to 1.9 fold) of CD44+/CD24- stem cells when compared to orthotopic mammary fat tumors (Figure 5B). The metastatic lung lesions became more enriched for stem cells with every round of serial re-implantation (90–95%). Lastly, BC cells with stably activated *Oct3/4* (GFP+) evade paclitaxel-induced deaths (IC50 for MDA-MB-231) [23] to survive and proliferate significantly better than their GFP- counterparts (Figure 5A). A possible mechanism of resistance to paclitaxel may be the upregulated ONSS transcriptome directed increase in proportion of intracellular β-tubulin or enhanced drug efflux via ABC transporters. Our short-term proliferation assay cannot interpret these findings for the MDA-MB-231 BC cell line in general, the effect of activated *Oct3/4* is clear in this comparison between GFP+ and GFP- cells. We agree that more clarity on mechanisms of chemoresistance such as apoptosis and senescence could be clarified by future studies, including clonogenicity assays, to better understand the mechanism of resistance in breast cancer. The increased tumorigenic potential of BC cells when the Oct3/4 gene is activated and the capability to turn on its network of associated genes as a response to external modulators such as paclitaxel may be an important mechanism of the chemotherapy resistance acquired by metastatic breast tumors. 

## 4. Materials and Methods

### 4.1. Cell Culture

MDA-MB-231 cells were purchased from American Type Culture Collection (Manassas, MA, USA). This cell line was established in 1973 as a pure epithelial line from metastatic pleural effusion from a breast cancer patient with a mean chromosome number between 60–70. The treatment of the patient included 5-FU, prednisone and combined systemic chemotherapy (cyclophosphamide, Adriamycin and aminopterine), without benefits for the patient was described by Cailleau et al., in 1974. Depending on the experiments, the cells were maintained in DMEM Dulbecco’s modified Eagle’s medium with fetal calf serum (FBS, 10%) and standard antibiotics Penicillin 100 U/mL; Streptomycin 100 mkg/mL (Cat #30-002-01, Corning, NY, USA). Xenografts were maintained on the dishes covered by gelatin in the original *medium developed for CSCs* (CSCM) consisting of the Dulbecco/F12 medium and supplemented with FBS (15%)), standard antibiotics, medium conditioned by primary cultured human foreskin fibroblasts (HFF: 30%) and G418 (400 U/mL), depending on the experimental conditions. All cultures were maintained at a constant temperature of 37 °C with a humidified atmosphere of 5% CO_2_. Cells were routinely tested for mycoplasma contamination.

### 4.2. Generation of Oct3/4-eGFP Transgenic MDA-MB-231 Cell Sublines and Clones by Stable Transfection

To establish MDA-MB-231 cell sublines that constitutively express the extended Green Fluorescent Protein (GFP) reporter, a natural protein expressed in jellyfish Aequoma Victoria, we used strategies reported earlier [15]. The plasmid-having eGFP reporter under control of the full 4.2 KB fragment of the human POU5F1/OCT3/4 promoter from genomic DNA was kindly donated by Dr. Wei Cui (Roslin Institute, UK). This promoter fragment spans from 3917 to +55 bp, relative to the transcriptional start site, and contains all four conserved cis-regions (CR1 to CR4) that are known to be important for the developmental and tissue-specific expression of the Pou5F1/Oct3/4 gene in human, bovine and mouse models, and for the resistance to G418 under the CMV promoter for selection. To estimate the initial values of cells capable to spontaneously activate eGFP in vitro and considering the rate of eGFP spontaneous silencing in GFP+ cells, polyclonal MDA-MB-231 cells were co-transfected with two plasmids simultaneously. Linearized the pOct3/4-eGFP-having neomycin resistance marker with GFP reporter and pCMV-mRFP (monomeric Red Fluorescent Protein) reporter. The transfection was performed by using Ca-precipitation kit (Promega, Madison, WI, USA), as the manufacturer prescribed. Stable transfectants were obtained by long-term culture in the presence of a neomycin antibiotic. Several independent individual clones of Oct3/4-eGFP+ cells have been established by different approaches.

### 4.3. Flow Cytometry-Based Cell Sorting (FACS) and Establishing Monoclonal Colonies

Co-transfected polyclonal cells that expressed both the neomycin resistance and two reporters, GFP and RFP, were designated as MDA-MB-231GR (Green Red), whereas monoclonal cells from clone #9 expressing eGFP were designated as MDA-MB-GFP+. Cells with the GFP+ fluorescence were trypsinized, suspended in phosphate-buffered saline (PBS) and isolated using FACSAria (BD, Bioscience, San Jose, CA, USA) to purify GFP+ and GFP- cell counterparts. The samples were first gated using SSC vs. FSC, and subsequently assessed for GFP+ and GFP- populations. Ten clones were isolated from MDA-MB-231GFP+ cultures by using different approaches combined with a selection of “Green” cells (MDA-MB-231 GFP+) or “non-green” cells, with silent expression, as shown by FACS.

Using multiple rounds of culture and selection by FACS, cells stably expressing GFP (designated MDA-MB-231GFP+) and cells stably expressing RFP+ fluorescence but the absence of GFP (designated as MDA-MB-231GFP-), were used to establish stable colonies. These stable colonies were used in all tumor growth studies, to establish our metastatic model and the proliferation assay. The expression of Oct3/4 was measured using FACS in these clones, as described in Supplementary Figure S1.

### 4.4. RNA Isolation and RT-PCR Analyses of Gene Expression

The total RNA was isolated by using the phenol/chloroform kit (RNA STAT- 60, Tel-Test Inc., Friendswood, TX, USA) according to the manufacturer’s instructions. cDNA synthesis was performed with 500 ng of the total RNA by using Superscript II (Invitrogen, Waltham, MA, USA) and Oligo(dt)12–18 (Invitrogen) according to the manufacturer’s instructions. The original primers for the selected genes were designed by using OLIGO5.0. All sequences were analyzed by BLAST search to ensure that they do not have significant sequence similarities with other genes.

### 4.5. Monoclonal Oncosphere Culture System Stem Cell Culture (SCC) Medium

Single-cell suspension from mouse tumor tissue or cultured cells were prepared according to the methods previously published [17,21]. Briefly, cells were plated at a density of 60,000 cells/well 6-well plastic dishes with an ultra-low attachment surface (Costar) in DMEM/F12 medium mixed with methylcellulose (final concentration 0.8%) and supplemented with progesterone (20 nM), putrescine (100 mkM), sodium selenite (30 nM), transferrin (25 mkg/mL), epidermal growth factor (10 ng/mL), fibroblast growth factor (10 ng/mL), FGF2 (10 ng/mL) and insulin (20 mkg/mL) (Stem cell Culture (SCC) medium). The cells incubated for 7–12 days and were monitored with inverted phase contrast microscopy (Nikon Eclipse TS100) using the image program SPOT 3.2.6 for the appearance of sphere-like cell cluster colonies.

### 4.6. Cell Proliferation Assay in the Presence of Paclitaxel

MDA-MB-231GFP+ and MDA-MB-231GFP- cells (see Materials and Methods, Section 4.2 and Section 4.3) were seeded in 100-mm dishes at a density of 1 × 10^6^ cells/10 mL. The cells were treated with 10 nM of Paclitaxel. The cells were trypsinized at 24, 48 and 72 h and stained with trypan blue. The number of viable cells was counted using a hemocytometer. The dose selection and timing was selected from the literature [23].

### 4.7. Generating Orthotopic Primary Tumors and Spontaneous Lung Metastases Using BALB/C Mouse Xenograft Model

#### 4.7.1. Primary Tumor Growth Model

All animal studies adhered to the protocols approved by The Institutional Animal Care and Use Committee of University of Tennessee for TI under CA138488 (10 September 2013). For the injection to the fat pad, eGFP+ and eGFP- cells were trypsinized and resuspended at a density of 100,000 cells in 100 uL 1XPBS, then slowly grafted into the mammary fat pad of 7-week-old female nude mice (*n* = 5–9, Jackson Laboratories, Bar Harbor, ME, USA) using a 27-gauge needle. 

#### 4.7.2. Lung Metastasis Model 

Female BALB/C nude mice 8–10 weeks of age were anesthetized via an intraperitoneal injection of ketamine (100 mg/kg) and xylazine (10 mg/kg). An incision was made on the chest to separate the subcutaneous tissues from the chest, and 50 K cells were injected into the mammary fat pad. The animals were monitored every 2 days for up to 70 days for tumor growth, at which time the animals were sacrificed and autopsied for the analysis of primary and metastatic lung tumors.

#### 4.7.3. Flow Cytometry and Single Cell Sorting of CD44+/CD24-Populations

The expression of surface proteins on cultured cells was measured with flow cytometry performed on the machine BD LSR II with the following antibodies:

Mouse allophycocyanin (APC), conjugate of anti-human CD44, and mouse phycoerythrin (PE) conjugate of anti-human CD24, as well as *p* (IgG2a, k) and APC (IgG2b k) mouse isotype controls. All antibodies were purchased from BD Biosciences, Franklin Lakes, NJ, USA, or Miltenyl Biotech, Auburn, CA, USA.

#### 4.7.4. Flow Cytometry Staining

Transfected MDA-MB-231 cells were cultured in T-75 flasks coated with gelatin and were detached by incubation with Versene 1× (Invitrogen Corporation, Carlsbad, CA, USA). PBS was added to the suspension and the cells were counted. A total of 1 × 10^6^ cells were washed twice with PBS containing 0.5% BSA (5 min at 1200 rpm, 4 °C), and then they were resuspended in 200 µL PBS (per 10^6^ cells). Antibody was then added, and the cells were incubated on ice in the dark for 45 min, followed by washing the antibody–cell mixture twice with 0.5% BSA. Finally, the antibody–cell mixture was resuspended in a final volume of 500 µL of PBS and assessed for expression by a flow cytometry analysis using a BD LSRII (BD Bioscience, Franklin Lakes, NJ, USA). 

#### 4.7.5. Microarray Gene Expression Analysis

The total RNA was isolated from the indicated samples by a phenol/chloroform extraction using RNA STAT-60 (Tel Test, Friendswood, TX, USA), followed by ethanol precipitation and resuspension in RNase-free water. The total RNA integrity of each sample was determined by an Agilent 2100 Bioanalyzer (Agilent, Palo Alto, CA, USA). First and second strand cDNA were synthesized from 15 ug of the total RNA using the SuperScript Double-Stranded cDNA synthesis Kit (Invitrogen, Carlsbad, CA, USA) and cRNA was synthesized and labelled with biotinylated UTP and CTP by in vitro transcription using the Bio array High Yield RNA Transcript Labeling Kit (ENZO Diagnostics Inc. Farmingdale, NY, USA), as per the manufacturers’ instructions. Labeled cRNA fragmentation, array hybridization, washing, staining with Streptavidin–Phycoerythrein conjugate (SAPE), and scanning were performed according to the manufacturer’s standard protocols (Affymetrix) using Affymetrix FS450 Fluidics stations and the Affymetrix GCS 3000. The global gene expression in MDA-MB-231 cells with expressed or silenced Oct3/4-eGFP was analyzed in triplicate RNA samples generated from three independent cultures using Human Genome-U133 Plus 2.0 Gene Chip™ arrays (Affymetrix, Santa Clara, CA, USA), which contain oligonucleotide probes representing more than 39,000 well-characterized human genes.

Microarray data were acquired with Affymetrix MAS 5.0 software (GCOS v1.4), CEL files underwent global background normalization and log2 transformation by robust multi-array averaging (RMA) [48]. The significant differential gene expression was determined by a Welch‘s *t*-test, using a *p*-value of <0.01 and a >2-fold expression change. Based on these criteria, 17 genes exhibited significant differential expression (Differentially Expressed Genes or DEGs, *p ≤* 0.0001–0.01) in the analyzed samples. The results were confirmed by real-time reverse transcription PCR.

Gene Ontology and Pathway analyses were performed in GeneSifter (VisX Labs, Seattle, WA, USA) using the amiGO database (The Gene Ontology Consortium; http://amigo.geneontology.org.) and the Kyoto Encyclopedia of Genes and Genomes (KEGG) database (www.genome.jp/kegg). The significant enrichment of specific Gene Ontology (GO) categories or KEGG pathways was estimated by hypergeometric tests (*p*-values ≤ 0.05) using the HG-U133 Plus 2.0 array content as the reference set.

#### 4.7.6. mRNA Synthesis and Labelling

The first- and second-strand cDNA was synthesized from 15 micrograms of total RNA using the SuperScript Double-Stranded cDNA synthesis Kit (Invitrogene, Waltham, MA, USA) and oligo-dt24-T7 (5′-GGC CAG TGA ATT GTA ATA CGA CTC ACT ATA GGG AGG CGG-3′) primer (PrOligo, Singapore), according to the manufacturer’s instructions. cRNA was synthesized and labelled with biotinylated UTP and CTP by in vitro transcription using the T7 promoter-coupled double-stranded cDNA as template and the Bio array TM High Yield TM RNA Transcript Labeling Kit (ENZO Diagnostics Inc. Farmingdale, NY, USA).

#### 4.7.7. Oligonucleotide Array Hybridization and Analysis

A synthesized mRNA pellet was resuspended in 10 uL of RNase-free water, and 10 ug was fragmented by ion-mediated hydrolysis at 95 °C for 35 min in 200 mM of Tris-acetate (pH 8.1), 500 of mM potassium acetate and 150 mM of magnesium acetate. The fragmented cRNA with the average sizes of 35–200 bases were hybridized for 16 hrs at 450 C to GeneChip Human Genome U133 Plus 2.0 arrays (Affymetrix), which cover over 47,000 human transcripts and variants, representing approximately 39,000 of the best characterized human genes (Affymetrix). The chips were processed on an automated fluidic station according to protocols provided by Affymetrix, including antibody amplification. After scanning the arrays on a Hewlett-Packard Gene Array scanner, data were acquired with Affymetrix MAS 5.0 software to analyze gene expression results. Briefly, the signal intensity for each gene was calculated after raw expression values for PM probes in the Affymetrix. CEL files underwent global background normalization and log2 transformation by robust multi-array averaging (RMA) [48]. Significant expression changes were determined by a Welch’s *t*-test, using a *p*-value of <0.01 and a >2-fold change as filtering criteria. Gene Ontology and Pathway analyses were performed in GeneSifter (VisX Labs) using the amiGO database (The Gene Consortium), and the metabolic pathway analysis was performed using the Kyoto Encyclopedia of Genes and Genomes (KEGG) database.

#### 4.7.8. Validation of Affimetrix Gene Chip Results by Using Real Time Semi-Quantitative Reverse Transcriptase PCR

The same RNA samples were used for microarrays and validation analyses. Ten genes demonstrating significant expression differences between high and low Oct3/4-eGFP expressing MD-231 cells were targeted. Suitable primers for the ten genes of interest were generated by using Oligo 5.0 software. Three-step semi-quantitative reverse transcriptase -PCR was performed for ten genes in ten tubes with 40 uL of the PCR master mix containing 500 ng of cDNA. A total of 10 uL probes were taken at intervals between 25, 30 and 35 cycles. GAPDH or beta-2-microglobulin were used to standardize the results.

#### 4.7.9. Quantitative Real-Time Reverse Transcription PCR Analyses

The total RNA was extracted using RNA-STAT-60 isolation kit (Tel-Test Inc., Friendswood, TX, USA). All samples of the total RNA were treated by DNAase I and checked for contamination of genomic DNA by PCR reaction with primers for Oct3/4B. The concentration of the total RNA was measured by Nanodrop (Roche, Nutley, NJ, USA). First Strand cDNA was synthesized from 1 mcg of the total RNA in a 20 uL reaction with oligo(dt) primers using the Transcriptor First Strand cDNA Synthesis kit (Roche). Quantitative real-time reverse transcription–PCR (qRT-PCR) was conducted with the Taq SYBR Green Light Cycler 480 Probe Master (Roche) using the LC480 Cycler System (Roche). Sequences of primers for RT-PCR may be available on demand. All primers were designed using the Universal Probe Library for Human (Roche). Relative expression levels were determined by using the ∆∆Ct (prizma) method with data from triplicate multiplexed reactions normalized to GAPDH. Data were analyzed by using the *t*-test and one-way Anova.

## 5. Conclusions

Our results fit well with the growing body of evidence associating breast cancer metastasis with genes and pathways involved in normal embryonic development. The *Oct3/4* regulated network primarily controls the plasticity and self-renewal of ES cells [49]. Genetic mechanisms of tumor initiation and development by these embryonic stem cells is yet to be fully understood. It may be possible that cancer stem cells use the machinery of self-renewal to promote and maintain tumorigenesis. Our results highlight a profile of PFs and other TFs with modulated expression, associated with a Oct3/4 activation that strongly correlates with increased BC tumorigenesis and metastasis. The upregulated transcriptome strongly correlates with a stem-cell-like phenotype of these cancer cells and demonstrates increased resistance to treatments such as paclitaxel. Further studies are needed to establish the causative effect of the *Oct3/4* network of associated genes in promoting tumorigenesis by using a system with *Oct3/4* overexpression and in vitro/ in vivo knock down models of key DEGs in the *Oct3/4* network. This will help to identify promising molecular targets in the BC metastatic cascade and thus develop effective treatment strategies.

## Data Availability

All data generated or analyzed during this study are included in this published article and its supplementary information files.

## Supplementary Materials

The supporting information can be downloaded at: https://www.mdpi.com/article/10.3390/ijms24119142/s1.
