# Supplementary material for: Upregulation of the Oct3/4 Network in Basal Breast Cancer Is Associated with Its Metastatic Potential and Shows Tissue Dependent Variability
Source: Int J Mol Sci. 2023 May 23;24(11):9142. doi: 10.3390/ijms24119142 (PMC10252816; doi:10.3390/ijms24119142)
Supplement: Supplementary file 1 [file ijms-24-09142-s001.zip › ijms-2301565-supplementary.pdf]

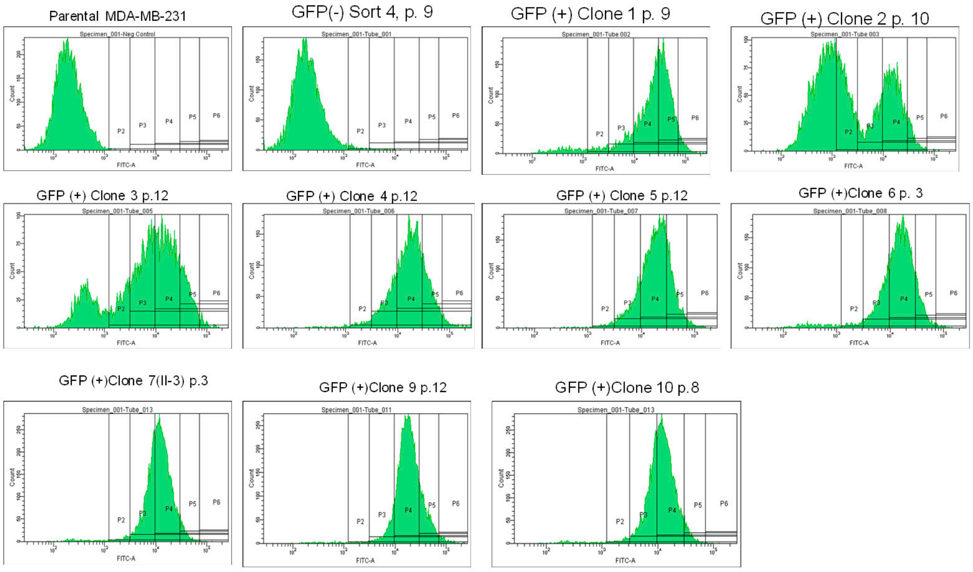

Clones	GFP^{+}	GFP^{+}	GFP^{+}	GFP^{++}	GFP^{+++}
	$P2 > 10^{3}$	$P3 > 10^{35}$	$P4 > 10^{4}$	$P5 > 10^{45}$	$P6 > 10^{6}$
Par	0.2	0	0	0	0
GFP	1.7	0.1	0	0	0
1	95.6	92.8	84.8	47.5	4.8
2	58.6	38.0	27.6	3.8	0.1
3	83.5	74.4	47.0	13.8	1.2
4	99.1	96.8	78.7	24.1	3.0
5	99.7	98.3	84.1	24.3	1.6
6	96.8	94.7	75.2	12.6	0.7
7	98.9	97.1	66.6	4.6	0.1
9	99.1	98.5	91.1	19.9	1.8
10	96.5	95.3	89.8	33.0	1.4

Supplemental Figure S1: MDA-MB-231 cell line transfected with *Oct3/4*-GFP were screened using GFP fluorescence with FACS. Selected GFP+ clones were cultured in conventional media and growth conditions and assessed for the stability and uniformity of GFP fluorescence as a measure of intra-clonal heterogeneity. (Par=Parental MDA-MB-231 Cell line)

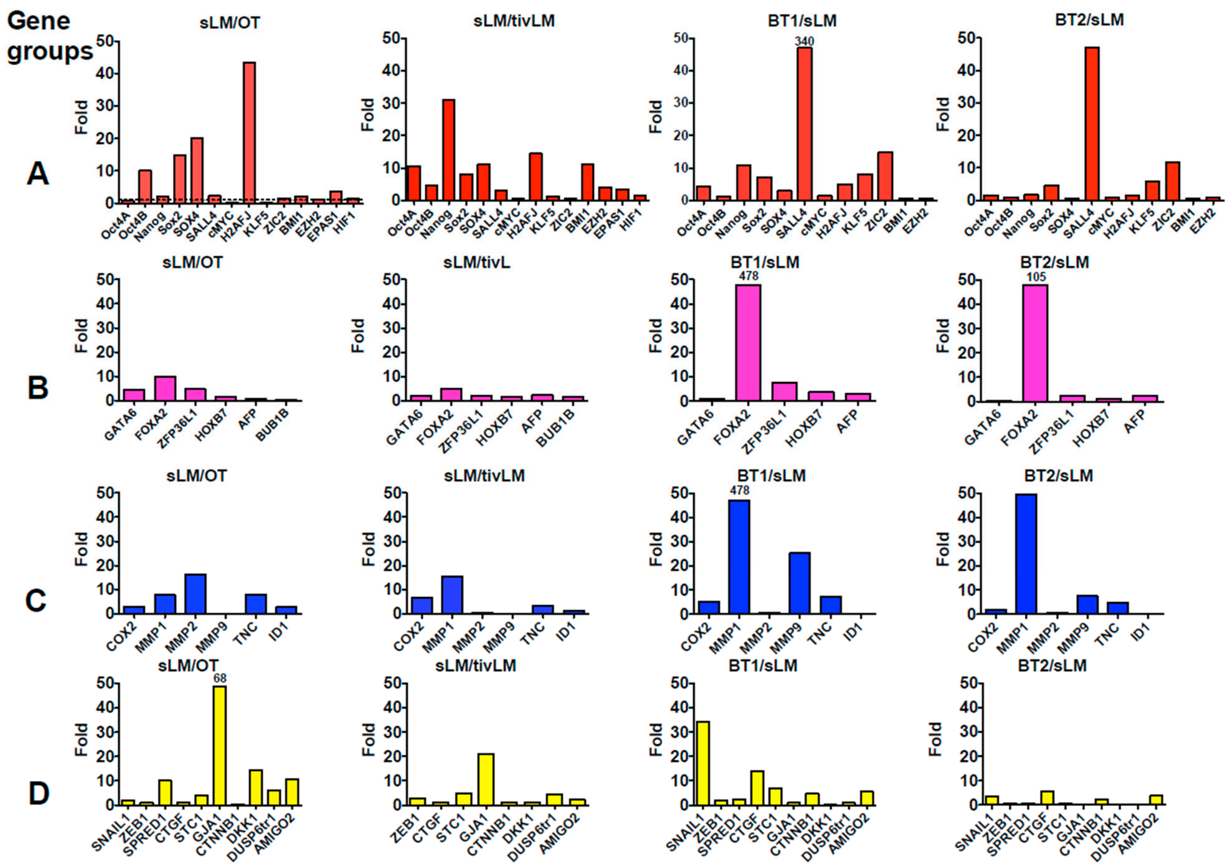

Supplemental Figure S2: Heterogeneity of Oct 3/4 GFP expression and related transcriptome within primary tumors and metastatic lesions in different tissues. qRT-PCR analysis of differentially expressed genes of the OCT3/4 network in the GFP+ cells compared to GFP- cells within primary tumors and metastasis in different tissues. Set of genes based on similarity in known functions are organized into Gene groups (A-D). Within each group (A-D), Fold changes of each gene is with respect to the housekeeping genes presented as a ratio of spontaneous lung metastasis (sLM), orthotopic (OT) and brain tumor directly implanted (BT1-BT2) and Tail vein injection of tumor cells establishing in Lungs (tivLM). This figure is supplement to figure 4 in the manuscript.
